# Self-Compassion Intervention Programs for Nurses: A Scoping Review

**DOI:** 10.3390/healthcare13020177

**Published:** 2025-01-17

**Authors:** Jing Bian, Fazhan Chen, Shihan Fang, Yanbo Wang

**Affiliations:** 1 School of Medicine, Tongji University, Shanghai 200124, China; 2241133@tongji.edu.cn (J.B.); 2241134@tongji.edu.cn (S.F.); 2 Clinical Research Center for Mental Disorders, Shanghai Pudong New Area Mental Health Center, School of Medicine, Tongji University, Shanghai 200124, China; develop909@163.com

**Keywords:** nurses, self-compassion, mindfulness, intervention

## Abstract

**Background**: Nurses frequently face various sources of stress in the workplace, making self-compassion interventions crucial for promoting their mental well-being. This scoping review aims to: (a) identify self-compassion intervention programs implemented within the nursing population; and (b) analyze the content and outcome measures of these interventions. **Methods**: The study follows Arksey and O’Malley’s scoping review framework and adheres to the PRISMA-ScR guidelines. Relevant literature on self-compassion interventions for nurses published between January 2010 and May 2024 was systematically reviewed. Databases searched included PubMed, Web of Science, Scopus, ProQuest, and the Cochrane Library, using MeSH terms and free-text keywords such as “self-compassion”, “self-kindness”, “self-appreciation”, “self-worth”, “self-forgiveness”, “self-awareness”, “nurses”, and “nursing”. **Results**: Fifteen studies met the inclusion criteria and were included in the review. Self-compassion interventions were categorized into two types: (1) mindfulness-focused programs, such as Mindfulness-Based Stress Reduction (MBSR) and Mindful Self-Care and Resiliency (MSCR), which treat self-compassion as a potential outcome; and (2) self-compassion-focused programs, including Mindful Self-Compassion (MSC), Compassion Focused Therapy (CFT), and Loving-Kindness Meditation (LKM). **Conclusions**: The mental health challenges faced by nurses have drawn growing attention, underscoring the importance of self-compassion interventions. This review examines empirical studies within the nursing population, contributing to the development of more targeted and effective strategies to enhance the mental health and well-being of nursing professionals.

## 1. Introduction

Nurses are a high-risk group for mental health disturbances, with a pressing need for improvement in their psychological well-being. In the workplace, unpleasant behavior [1] horizontal violence [2], patient safety incidents [3], and other stressors are commonplace for nurses. As positive psychology gains prominence, emphasizing the use of personal strengths to combat negative emotions, self-compassion has garnered increasing attention as a crucial protective factor for mental health.

Numerous studies have linked self-compassion to reduced procrastination [4], post-traumatic growth [5], resilience to occupational risk [6], and enhanced subjective well-being [7]. Broadly speaking, self-compassion serves as a critical protective factor for mental health and has been validated as effective in diverse populations, including adolescents, individuals with psychological disorders, and patients with chronic illnesses. Among healthcare professionals, self-compassion interventions have proven to be an effective strategy for addressing secondary traumatic stress, and occupational burnout [8], helping to alleviate empathy fatigue and work-related stress [9]. The emotional health of professionals directly impacts the quality of care and services they provide within the health system. In the face of distress, self-compassion is essential for nurses’ self-care and well-being and is closely linked to their compassion for others [10].

Despite the obvious potential benefits, self-compassion may not come naturally to many nurses. While many nurses may be inclined to hold critical or judgmental attitudes towards themselves, particularly after clinical errors or perceived failures, this self-criticism can be counterproductive to their well-being and professional performance [11]. A constructivist study suggests that in a stressful and fast-paced work environment, nurses need explicit permission and encouragement to engage in self-care and self-compassion [12]. Importantly, self-compassion is not an inherent personality trait but a skill that can be learned and cultivated [13,14]. Therefore, given the high levels of stress in the nursing profession, developing self-compassion is essential for coping with workplace challenges and maintaining both personal well-being and the quality of patient care.

Self-compassion, first introduced by American psychologist Neff in 2003 [15], refers to an individual’s ability to offer themselves support during times of distress, whether stemming from personal mistakes, shortcomings, or external life challenges. It encompasses three dimensions: self-kindness, common humanity, and mindfulness [13]. Self-kindness represents the emotional aspect of self-compassion, involving understanding and forgiveness toward one’s own flaws and imperfections; common humanity reflects the cognitive dimension, emphasizing the acceptance of human imperfection—that everyone experiences failure and makes mistakes. This perspective fosters a sense of connection with others rather than isolating oneself in adversity. Mindfulness pertains to the attentional component, characterized by a clear and balanced awareness of the present situation. It avoids either ignoring or obsessing over negative aspects of oneself or life [16]. Self-compassion involves internalizing the compassion one typically extends to others. The Dalai Lama has pointed out that to generate genuine compassion for others, one must have a foundation for cultivating compassion, which is the ability to connect with one’s own feelings and care for one’s well-being [10].

Given the positive impact of self-compassion on mental health, an increasing number of scholars are focusing on cultivating and enhancing self-compassion levels. Research on self-compassion among healthcare professionals primarily explores its influence on the quality of professional life. Most studies adopt a cross-sectional design, investigating the relationships between variables and examining self-compassion either as an independent or mediating variable affecting outcome variables. However, intervention studies remain relatively scarce [17]. Systematic reviews of interventions aimed at improving the mental health of healthcare professionals indicate that most strategies focus on enhancing mindfulness levels, with relatively few studies targeting self-compassion as the primary intervention objective [18,19]. In terms of intervention formats, beyond traditional in-person psychological interventions, Fainstad et al. explored the use of online group counseling interventions [20]. However, these approaches faced challenges such as small sample sizes and high attrition rates [21]. Moreover, self-compassion interventions are typically conducted over eight weeks; however, Bluth et al. suggested that a six-week intervention might achieve higher acceptance among nurses while maintaining comparable effectiveness [22]. Consequently, the content, format, outcome measures, and efficacy of self-compassion interventions in nursing populations exhibit considerable heterogeneity, and their effectiveness requires further validation through high-quality research.

The purpose of this scoping review is to map existing evidence on self-compassion interventions and strategies in nursing, with a focus on summarizing intervention content to inform future research aimed at enhancing self-compassion among nurses.

## 2. Methods

A scoping review is a method of summarizing evidence based on the concept of evidence-based practice, which can quickly help researchers identify the progress of research in a particular area and the sources and types of evidence available, summarize research findings, and identify existing problems in research [23].

The methodological framework developed by Arksey and O’Malley [24] outlines a five-stage process: (a) identify the research question; (b) identify relevant studies; (c) select studies; (d) chart the data; and (e) summarize and report the results. The results were reported following the PRISMA-ScR(Preferred Reporting Items for Systematic Reviews and Meta-Analyses Extension for Scoping Reviews) checklist, providing a reference point for clinical healthcare professionals to implement self-compassionate interventions and conduct related research. The protocol for this review was published in the OSF Registry (https://osf.io/xqnje, accessed on 5 December 2024), with the DOI 10.17605/OSF.IO/XQNJE.

### 2.1. Identifying Research Questions

This scoping review aims to explore, examine and understand the following:

1. What are the intervention programs of self-compassion for nurses?
2. What are the components of the self-compassion intervention program, and how have the included studies reported on its measurement and effectiveness?

### 2.2. Identifying Relevant Studies

The search strategy encompassed five online databases: PubMed, Web of Science, Scopus, ProQuest, and Cochrane Library. The search period covered 1 January 2010–29 May 2024, related to the designated search terms. The rationale for selecting this timeframe is that beginning in 2010, research on self-compassion experienced a marked increase [13].

Keywords were selected using a combination of MeSH terms, free-text terms and Truncation including “self-compassion”, “self-forgiveness”, “self-kindness”, “nurse” and their variants, which were combined using AND/OR. A title and abstract screening was conducted to ensure that the included studies were relevant to the research questions. Only full-text articles published in English were included in the search. A complete search strategy was developed, utilizing text words from relevant articles’ titles and abstracts, along with index terms used for article descriptions. The detailed search terms and strategy can be found in Table 1.

### 2.3. Study Selection

The retrieved documents were imported into EndNote 21 software to screen for duplicate screening, and two trained researchers independently reviewed the titles and abstracts for initial screening based on the inclusion and exclusion criteria (Table 2), followed by full-text review for a second screening. In case of disagreement during the screening process, a third researcher was consulted to discuss and decide on the inclusion of the literature. The literature that met the criteria was identified. Data were independently extracted by two researchers.

### 2.4. Charting the Data

**Figure 1:**
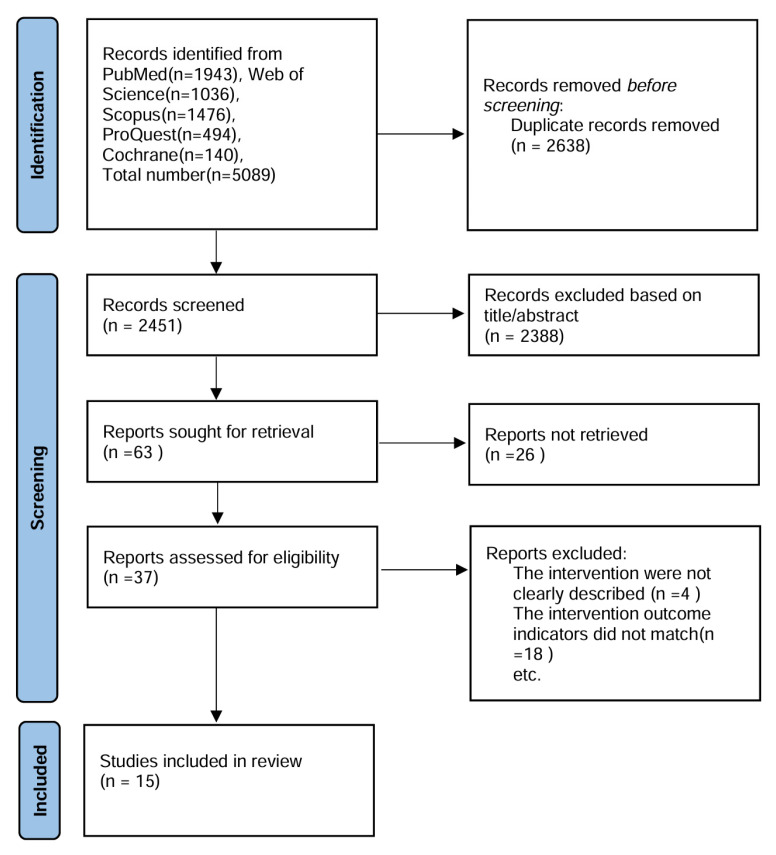
PRISMA diagram of search strategy.

A PRISMA flow diagram clearly delineates the article selection process (Figure 1). The quality of the research is assessed using a standard quality evaluation tool, which is employed to evaluate both quantitative and qualitative studies. Each criterion is scored as follows: 0 (not addressed), 1 (partially addressed), or 2 (fully addressed). The total score is converted into a percentage using the method proposed by Kmet et al. [25]. All studies included in this review scored above 60%, which is considered a reasonable threshold by the authors of the tool. The research in this study is evaluated and scored according to the quantitative research quality assessment checklist, with an average score exceeding 86.4% (see Table 3). Of the articles retained for analysis, the authors used a data abstraction form to identify variables such as the title, author, country, date of publication, study population, study type, sample size, intervention method, intervention content, intervention duration, outcome indicators, and measurement tools of the trial and control groups.

## 3. Results

### 3.1. Basic Characteristics of the Included Literature

A total of 5089 articles were initially retrieved. After removing duplicates, 2451 articles remained. Titles and abstracts were screened, leading to the exclusion of 2414 articles. A full-text review was conducted for further screening, resulting in the exclusion of four articles due to unclear descriptions of the intervention content, and 18 studies were excluded for not using self-compassion as an outcome measure. In total, 22 articles were excluded, leaving 15 articles for inclusion in the final review [26,27,28,29,30,31,32,33,34,35,36,37,38,39]. The included studies comprised two randomized controlled trials [26,40], 11 quasi-experimental studies [27,29,30,31,32,33,35,36,37,38,39], one mixed-methods study [33], and one case study [28].

The included studies were conducted in a variety of countries, showcasing a balance between Western and Eastern regions. Specifically, studies were carried out in the United States (n = 4) [26,34,35,40], the United Kingdom, Canada, and Ireland (one study each) [33,37,39], representing Western nations. From the Eastern regions, studies were conducted in China (n = 2) [28,29], India, and Egypt (one study each) [30,31]. Additional contributions came from Australia, Portugal, and Finland (one study each) [27,32,36]. The articles retained for analysis are presented in a table, which includes the title, authors, country, publication date, study population, study type, sample size, intervention methods, intervention content, duration of intervention, and research outcomes (see Table 4).

### 3.2. Synthesis of Study Findings

#### 3.2.1. Self-Compassion Intervention Content

The review results indicate that self-compassion interventions primarily fall into two categories. One category targets mindfulness as the intervention focus, with self-compassion as the underlying goal. Typical intervention programs include Mindfulness-Based Stress Reduction (MBSR) and Mindfulness-Based Compassionate Resilience (MSCR). The other category focuses on self-compassion, with typical programs including Mindful Self-Compassion (MSC), Compassion-Focused Therapy (CFT), and Loving-Kindness Meditation (LKM). Other interventions primarily focus on emotional regulation. Mindfulness refers to consciously and purposefully attending to present-moment experiences without judgment. Mindfulness-based interventions typically embrace a wide range of experiences, including both positive and neutral states. Mindfulness interventions are widely used in mental health to improve physiological and psychological well-being, as well as job satisfaction among individuals [41]. Numerous studies indicate that mindfulness can alleviate anxiety and depressive symptoms, while also improving self-compassion and emotional regulation [42,43]. However, some studies suggest that self-compassion may surpass mindfulness in alleviating emotional distress. Neff derived the idea of self-compassion from the broader Buddhist concept of compassion toward others [15], proposing that recognizing one’s self-compassion is strengthened by reflecting on the compassion one shows to others. Self-compassion interventions emphasize recognizing personal distress, acknowledging our shared humanity, and practising self-soothing. It may provide a stronger buffer against stress responses than mindfulness alone [44]. In clinical settings, a growing body of research increasingly emphasizes self-compassion within mindfulness interventions [45].

The intervention components of the fifteen included studies primarily included mindfulness, compassion, emotional intelligence, compassionate meditation, and music. Seven studies [27,29,31,32,34,36,38] included mindfulness elements. Adapted from Mindfulness-Based Stress Reduction (MBSR) [46] developed by Jon Kabat-Zinn in 1979, was employed to aid patients in managing stress, pain, and illness. MBSR has been extensively applied across diverse sectors, including healthcare, education, and the judiciary, with a focus on fostering mindfulness. The purpose of the mindfulness-based interventions was to cultivate present-moment awareness among participants through both formal and informal meditation practices. These included an introduction to mindfulness theory, various forms of mindfulness meditation (focusing on breath, body, mind, and movement), and mindfulness communication. Specific practices taught included sultana exercises, breath-anchored meditation, body-scanning meditation, three-minute breathing space, and mindfulness walking. Different mindfulness training techniques yield distinct outcomes. Guided Respiratory Mindfulness Therapy (GRMT) is a breath-focused mindfulness intervention that emphasizes breath regulation and mindfulness of bodily sensations in a relaxed setting. This method allows for the experiencing of both positive physical and mental states as well as challenging encounters with previously avoided negative thoughts, emotions, and traumatic memories, leading to profound relaxation and emotional integration. A comparative study in a caregiving environment demonstrated that both GRMT and other Mindfulness-Based Interventions (MBI) significantly enhance mindfulness and self-compassion, but GRMT proved more effective in reducing stress [29].

Three studies [33,35,37] reference Mindful Self-Compassion (MSC) training developed by Neff, a leading researcher in self-compassion, and Germer, a psychologist from Harvard [47]. MSC training includes teaching the principles of compassion and self-compassion, compassion meditation, mindfulness exercises, fostering self-compassionate awareness, managing difficult emotions, and accepting one’s current life phase. The training incorporates practices such as a supportive touch, crafting a personal loving-kindness phrase, writing compassionate letters to oneself, recording life’s positive events, and maintaining a gratitude journal. One study [35] involved a one-day self-compassion training program, known as Self-Compassion for Healthcare Community (SCHC), which was adapted from the MSC program. This program replaced the formal meditation practices of Mindful Self-Compassion (MSC) with informal exercises, such as placing a hand on one’s heart and offering kind words to oneself during challenging moments [48]. This adaptation was designed to accommodate the high-intensity work pace of healthcare professionals, allowing them to practice self-compassion in the workplace. The study indicated that healthcare professionals benefited more from informal exercises than from formal practices [48,49].

Additionally, one study [39] utilized Compassion Focused Therapy (CFT) with interventions that included compassionate education, compassionate imagery, developing a compassionate self, and fostering greater compassion. Valluri et al. [26] used Loving-Kindness Meditation (LKM) as the intervention, which included loving-kindness meditation and micro-practices. Sawyer et al. [40] developed an intervention program (RISE) based on four themes: resilience, insight, self-compassion, and empowerment. Saikia et al. [30] focused on emotional intelligence as the intervention content, covering its concepts, underlying neuroscience mechanisms, and clinical applications. Shum et al. [28] utilized Guided Imagery and Music (GIM), which differs from other self-compassion training methods by incorporating music. This technique, which engages the conscious state and focuses on the present moment, explores consciousness through images spontaneously generated by participants while listening to music.

#### 3.2.2. Forms of Self-Compassion Intervention

The self-compassion interventions were conducted through online, offline, and hybrid models, primarily involving classroom lectures, experiential and group exercises, the use of mindfulness apps, homework assignments, instructional videos on CD-ROM, and reflective writing. Ten [27,28,29,30,33,34,35,36,37,40] employed an offline training approach, providing participants with self-compassion sessions and experiential exercises. Two studies [31,32] utilized a mixed training model, where participants attended offline self-compassion sessions and were also instructed to download relevant apps for daily practice. Three studies [26,38,39] implemented an online intervention program, training participants in self-compassion through mobile apps.

The duration of self-compassion interventions was tailored to the specifics of the training content. Four studies [31,33,37,40] implemented an 8-week training period, aligning with the duration typically required for traditional interventions. Several studies adjusted the training lengths to better suit the demands of the workplace and the unique needs of healthcare settings: six weeks [32,36], 5 weeks [29], 4 weeks [38,39], and 3 weeks [29] in different studies, respectively. Franco et al. [35] condensed the SCHC intervention into a single day, totaling 6 h, to evaluate whether a one-day session could enhance resilience, well-being, and professional quality of life among pediatric nurses. Gauthier et al. [34] adapted an MBSR program into a concise format that involved 5 min of mindfulness meditation daily, altering the meditation focus every three days over a 30-day period. Boch et al. [38] facilitated self-help exercises using the Clam app, directing participants to engage in 10 min of mindfulness meditation daily for 4 weeks Slatyer et al. [27] offered a one-day training in Mindful Stress Reduction Therapy, consisting of four sessions, each 1.5 h long, followed by three weekly 1.75-h sessions of mindful practice maintained over three weeks. Shum et al. [28] conducted a year-long case study involving 21 sessions, each ranging from 90 to 120 min. This illustrates that there is considerable variability in the frequency and duration of self-compassion interventions.

#### 3.2.3. Self-Compassionate Outcome Indicators and Effects

The evaluation of the intervention’s effectiveness included assessments of nurses’ mindfulness level, self-compassion, burnout, compassion fatigue, subjective well-being, psychological resilience, nursing stress, and chronic pain. The results indicated varying degrees of improvement across all these aspects. The three-month follow-up results indicated that the effects of the intervention were largely maintained after its conclusion [27,35]. Self-compassion interventions have potential long-term benefits.

Positive psychological outcomes were measured using tools such as the Five Facets of Mindfulness Questionnaire (FFMQ), the Mindful Attention Awareness Scale (MAAS), the Self-Compassion Scale (SCS) or its Short Form (SCS-SF). Negative emotional aspects were primarily evaluated using the Professional Quality of Life Scale (ProQOL-5), the Depression, Anxiety, and Stress Scale (DASS-21), and the Nursing Stress Scale (NSS).(see Table 5).

## 4. Discussion

### 4.1. The Content of Self-Compassion Interventions for Nurses Is Currently Limited and Requires Further Expansion

Although the types and durations of interventions varied across studies, the central focus remained on cultivating self-compassion. The included studies consistently demonstrated that self-compassion interventions positively impact the psychosocial factors of nurses’ workplaces and are generally well-accepted.

Currently, self-compassion interventions for nurses primarily focus on enhancing participants’ mindfulness levels, with self-compassion being assessed as an outcome measure. Most interventions currently focus on mindfulness meditation, with a few studies exploring the use of music, mandala imagery, and emotional intelligence exercises. As research on self-compassion deepens, studies targeting self-compassion specifically have become more prevalent. This study highlights that research focusing on self-compassion interventions within the nursing population is limited. It is noteworthy that the study by Brito-Pons et al. [14] found that although both types of interventions improved psychological well-being and increased self-compassion, interventions targeting self-compassion had a greater impact on developing self-compassion skills. Additionally, some research has induced self-compassion experimentally to explore its effects on psychological and physiological stress recovery [50]. Numerous studies have shown that healthcare workers experience high levels of compassion fatigue [51,52,53]. Training in self-compassion may serve as a crucial intervention to reduce burnout and compassion fatigue [54]. Self-compassion interventions can provide protective factors against compassion fatigue and enhance nurses’ psychological resilience [33]. To advance mental health care, future research should effectively incorporate compassion science and expand intervention methods, such as music therapy and experimental induction, to explore the impact of both external interventions and internal inductions on self-compassion. This approach could better enhance nurses’ well-being and improve patient outcomes.

### 4.2. Greater Heterogeneity in the Frequency and Duration of Self-Compassion Interventions for Nurses

There is considerable variability in the frequency and duration of self-compassion interventions for nurses, with intervention lengths ranging from three weeks to twelve months. Some studies implemented interventions over four weeks, while others extended them to eight weeks. The frequency of interventions was typically once per week, though some studies required up to three sessions per week depending on the context, while others did not specify a frequency, allowing participants to engage with the program at their discretion. Interventions that lack an evidence-based approach to frequency and duration may fail to provide nurses with effective self-compassion training. Since self-compassion is a trait that can be developed through training, the determination of appropriate training duration and frequency is crucial in achieving optimal outcomes.

The adherence and participation rates in various studies differ significantly. Although online interventions generally enjoy higher acceptance, they still face challenges such as high dropout rates [32,39], possibly due to a mismatch between the time investment required and the perceived immediate benefits, as well as individual attitudes toward psychological training and self-compassion. Some researchers have condensed training into a single day or integrated it into nurses’ morning and evening handovers to accommodate their clinical work schedules. Studies indicate that a one-day self-compassion intervention can have positive effects on resilience, emotional well-being, and reducing burnout, anxiety, and stress, with participants maintaining these improvements at a three-month follow-up, suggesting the intervention’s lasting impact [35]. Nonetheless, the study by Gauthier et al. [34], which implemented a daily 5-min mindfulness meditation, found no significant effects on enhancing mindfulness and self-compassion. This lack of impact may be related to the short duration of the intervention. Integrating self-compassion interventions into clinical practice remains a crucial area for further research. Therefore, future studies should conduct high-quality randomized controlled trials to explore the optimal frequency and duration of interventions, determining the level of participation required to achieve positive outcomes.

### 4.3. The Evaluation Indicators for Nurses’ Self-Compassion Are Relatively Homogenous, Primarily Relying on Self-Reported Outcomes

The effectiveness of these interventions is mostly evaluated through self-reported measures of self-compassion-related outcomes, often serving as the sole metric, with a lack of external evaluations from instructors, peers, and patients. While enhancing nurses’ self-compassion is a typical objective and key outcome of effective training programs, the ultimate goal in clinical practice is not only to foster compassion and understanding toward oneself but also to facilitate the delivery of compassionate care and improve patient outcomes. Nevertheless, most self-compassion training interventions have not been evaluated from the patient’s perspective. As a form of positive psychological capital, self-compassion has been shown to correlate with a greater tendency to adopt positive coping strategies. Yet, few studies have included the impact of self-compassion interventions on coping with various workplace stressors as an evaluation criterion. Future research should incorporate qualitative studies to explore the effects of self-compassion training on patient outcomes and workplace coping strategies, addressing the limitations of quantitative research.

In terms of acceptability, only one study included in the literature employed “pulse surveys” during the intervention to collect real-time feedback from participants [32]. Most studies assessed the acceptability of the intervention through questionnaires, which may be subject to bias and lack objective indicators such as physiological or biochemical measures. In the future, wearable devices and smartphones could be used to implement ecological momentary assessment, allowing for repeated sampling of participants’ emotions or experiences during the intervention. This approach would provide real-time data that more accurately reflects the true state, offering a comprehensive evaluation of the effectiveness of self-compassion interventions among nurses.

### 4.4. Limitations

Firstly, this review only included studies in which nurses were the intervention subjects; studies on other health professionals were not included, and there may be some bias in the analysis of the findings. Secondly, since this study only included literature in English and Chinese, lacked large-sample randomized controlled trials, and did not assess the quality of the included studies, these limitations reduce the strength of the evidence.

## 5. Conclusions

This study summarizes the content, frequency, duration, outcome indicators, and effectiveness evaluation of self-compassion interventions for nurses. Self-compassion interventions are often integrated with mindfulness practices, which effectively enhance both mindfulness and self-compassion, reduce burnout, and improve work well-being. Compassionate nursing is increasingly recognized for its importance, particularly in the context of humanistic care in healthcare settings. Both compassion for others and self-compassion are essential, especially in the nursing profession, where fostering self-compassion helps to deliver compassionate care and improve patient outcomes. Currently, most self-compassion interventions are conducted in group settings. However, with the rise of artificial intelligence, it is suggested that future interventions could leverage AI technologies, such as big data analytics and user profiling, to provide personalized self-compassion support for nurses.

## Figures and Tables

**Table 1:** Search strategy.

Concept	Key Words or Synonyms
Nurse	“Nurses” OR “Nursing Personnel” OR “Personnel, Nursing” OR “Registered Nurses” OR “nurs*”
Self-Compassion	“Self-compassion” OR “Self-forgiveness*” OR “Self-forgiveness” OR “self-compassion*” OR “self-compassion” OR “self-kindness*” OR “self-kindness” OR “self-worth*” OR “self?worth” OR “self-appreciation*” OR “self?appreciation” OR “self-awareness*” OR “self?awareness”
Search Logic	“nurse” AND “self-compassion”
Databases	PubMed, Web of Science, Scopus, ProQuest, Cochrane Library
Search Field	title, abstract, keyword
Conducted Time	January 2010~May 2024

“*”: truncator.

**Table 2:** Eligibility criteria.

Inclusion Criteria	Exclusion Criteria
Study subjects were nurses, including midwives and other nursing professionals Study design including randomized controlled trials, quasi-experimental studies, mixed method or case studies Strategies or Intervention content was clearly defined.	Inaccessible full texts Duplicate publications Conference abstracts, research protocols, reviews, systematic evaluations, guidelines, opinion pieces, and policy documents non-English literature

**Table 3:** Critical appraisal of reviewed studies.

Quantitative Studies	Valluri et al. [26] 2024	Slatyer et al. [27] 2018	Shum et al. [28] 2020	ShuChen et al. [29] 2023	Saikia et al. [30] 2022	Sahar et al. [31] 2023	Mäkinen et al. [32] 2024	Martin et al. [33] 2018	Gauthier et al. [34] 2015	Franco et al. [35] 2021	Duart et al. [36] 2016	Crandall et al. [37] 2022	Boch et al. [38] 2024	Corrigan et al. [39] 2024	Sawyer et al. [40]. 2023
1: Question/objective sufficiently described?	Y	Y	Y	Y	Y	Y	Y	Y	Y	Y	Y	Y	Y	Y	Y
2: Study design evident and appropriate?	Y	Y	Y	Y	Y	Y	Y	Y	Y	Y	Y	Y	Y	Y	Y
3: Method of subject/comparison group selection or source of information/input variables described and appropriate?	Y	Y	Y	Y	Y	Y	Y	Y	Y	Y	Y	Y	Y	Y	Y
4: Subject (and comparison group, if applicable) characteristics sufficiently described?	Y	Y	N/A	Y	P	Y	Y	Y	Y	Y	Y	Y	P	P	Y
5: If interventional and random allocation were possible, was it described?	Y	N	N/A	N	N/A	N/A	N/A	N/A	N/A	N	N	N/A	N/A	N/A	Y
6: If interventional and blinding of investigators was possible, was it reported?	Y	N/A	N/A	N/A	N/A	N/A	N/A	N/A	N/A	N/A	N/A	N/A	N/A	N/A	Y
7: If interventional and blinding of subjects was possible, was it reported?	Y	N	N/A	N/A	N/A	N/A	N/A	N/A	N/A	N/A	N/A	N/A	N/A	N/A	Y
8: Outcome and (if applicable) exposure measure(s) well defined and robust to measurement/misclassification bias? Means of assessment reported?	Y	Y	Y	Y	Y	Y	Y	Y	Y	Y	Y	Y	Y	Y	Y
9: Sample size appropriate?	Y	Y	N/A	Y	Y	Y	Y	Y	Y	Y	Y	Y	Y	P	Y
10: Analytic methods described/justified and appropriate?	Y	Y	Y	Y	Y	Y	Y	Y	Y	Y	Y	Y	Y	P	Y
11: Some estimate of variance is reported for the main results?	N	Y	Y	Y	P	N	N	Y	P	Y	Y	Y	N	N	Y
12: Controlled for confounding?	P	P	N/A	P	P	P	N	P	P	P	P	P	P	N	P
13: Results reported in sufficient detail?	Y	Y	Y	Y	P	Y	Y	Y	Y	Y	Y	Y	Y	Y	Y
14: Conclusions supported by the results?	Y	Y	Y	Y	P	Y	Y	Y	Y	Y	Y	Y	Y	Y	Y
score (%)	25/28 89%	21/26 81%	16/16 100%	21/24 88%	17/22 77%	21/24 88%	18/22 82%	21/22 95%	20/22 91%	21/24 88%	21/24 88%	21/22 95%	18/22 82%	15/22 68%	27/28
															96%

YES (2) = Y; PARTIAL (1) = P; NO (0) = N; N/A = not applicable.

**Table 4:** Key characteristics of included studies (n = 15).

Author/Year/Country	Objective	Sample	Design	Interventions	Duration	Main Findings
Valluri et al. [26]/2024/United States	To assess the effectiveness of loving-kindness micro-practices on outcomes including chronic pain, stress, pulse rate, analgesic use, self-compassion, professional caring, and serum cortisol levels in nurses.	40 nurses	double-blind, randomized controlled pilot study.	The LKM group practised loving-kindness meditation and micro-practices (e.g., Serenity Pause, mindful breaths) daily, while the ACG group listened to TED talks and practised self-care as suggested in those lectures.	3 weeks	The study found no statistically significant differences between the LKM and ACG groups for most outcomes. However, self-reports indicated improvements in pain, anxiety, sleep, and stress reduction. Cortisol levels and analgesic use showed no significant changes.
Slatyer et al. [27]/2018/Australia	To assess the effectiveness of a brief Mindful Self-Care and Resiliency (MSCR) intervention in reducing burnout, secondary traumatic stress, and symptoms of psychological distress among nurses, and to examine its impact on compassion satisfaction, self-compassion, and resilience.	91 nurses	pre-test/post-test Quasi-experimental	The MSCR intervention involved a 1-day workshop followed by three weekly mindfulness practice sessions (total duration: 11.5 h). The program included education on compassion fatigue resiliency and mindfulness practices.	1-day workshop followed by three weekly sessions	The intervention group showed significant reductions in burnout and depressive symptoms, with these improvements persisting at the 6-month follow-up. Additionally, increases in compassion satisfaction, self-compassion, and subjective quality of life were observed.
Shum et al. [28]/2020/China	To evaluate the effectiveness of the Bonny Method of Guided Imagery and Music (GIM) in fostering self-compassion and enhancing psychological well-being in mental health nurses.	1 mental health nurse	Case study	Bonny Method of Guided Imagery and Music (GIM) sessions focus on self-compassion and psychological well-being. Each session included prelude, induction, music experience, and postlude phases.	12 months	The GIM intervention resulted in a 25.9% increase in the participant’s self-compassion score (SCS).
Sahar et al. [31]/2023/Egypt	To examine the effectiveness of mindfulness-based interventions (MBIs) in reducing burnout, enhancing mindfulness, and fostering self-compassion among critical care nurses (CCNs) caring for COVID-19 patients.	60 nurses (30 in the intervention group and 30 in the control group)	Quasi-experimental pre-test/post-test	The intervention group participated in eight 2.5-h mindfulness-based sessions over two months, while the control group received no intervention. The MBI was based on Mindfulness-Based Stress Reduction (MBSR) and Self-Compassion Training for Healthcare Communities (SCHC).	8 weeks	The study found that MBIs significantly reduced emotional exhaustion and depersonalization while increasing personal accomplishment, mindfulness, and self-compassion in the intervention group compared to the control group. These effects were statistically significant, with large effect sizes.
Mäkinen et al. [32]/2024/Finland	To determine the effects of mindfulness and self-compassion skills on stress and work satisfaction among emergency department (ED) and intermediate care unit employees, and to assess the feasibility of implementing the training during working hours.	49 nurses	Pre- and post-intervention study	The intervention consisted of two parallel components: individual mindfulness exercises via the mobile app (Andas Life) and six 1.5-h in-person group sessions. The individual exercises were based on Mindfulness-Based Stress Reduction (MBSR) and Mindfulness-Based Cognitive Therapy (MBCT).	6 weeks	The study found that instructor-led mindfulness training, combined with regular use of a smartphone mindfulness app, significantly reduced stress and burnout, while enhancing mindfulness and well-being among emergency department (ED) and intermediate care unit personnel.
Martin et al. [33]/2018/Ireland	To evaluate the impact of an eight-week pilot mindful self-compassion (MSC) training program on nurses’ compassion fatigue and resilience.	13 nurses	Observational mixed research pilot study	Eight-week MSC training program, including weekly 2.5-h sessions and a half-day retreat.	8 weeks	Significant reductions in secondary trauma and burnout, as well as increases in resilience and compassion satisfaction.
Gauthier et al. [34]/2015/United States	To explore the feasibility and effectiveness of a brief on-the-job mindfulness intervention for PICU nurses.	38 nurses	Pre-test/Post-test Pilot study	A 5-min mindfulness meditation before each work shift over a month.	30 days	Significant decreases in stress and burnout; non-significant increases in mindfulness and self-compassion over time.
Franco et al. [35]/2021/United States	To evaluate the effectiveness of a one-day self-compassion training for pediatric nurses.	53 nurses	Quasi-experimental pre- test and post-test	One-day self-compassion training workshop.	1 day	Significant increases in self-compassion, mindfulness, and resilience; decreases in burnout, anxiety, and stress.
Duarte et al. [36]/2016/Portugal	To explore the effectiveness of an on-site, abbreviated mindfulness-based intervention for oncology nurses.	94 nurses	Non-randomized, wait-list comparison design	A 6-week mindfulness-based group intervention based on Mindfulness-Based Stress Reduction (MBSR) principles	6 weeks	Significant decreases in compassion fatigue, burnout, stress, and experiential avoidance, along with increases in life satisfaction, mindfulness, and self-compassion among the intervention group. Medium to large effect sizes were observed.
Crandall et al. [37]/2022/Canada	To explore the effects of an 8-week Mindful Self-Compassion (MSC) training on nephrology nurses’ levels of self-compassion, burnout, and resilience.	12 nephrology nurses	Mixed methods study pre-post design	An 8-week MSC course involving meditation, experiential exercises, and group discussion.	8 weeks	Increases in self-compassion, mindfulness, and resilience, along with decreases in burnout and emotional exhaustion.
Boch et al. [38]/2024/United States	To investigate the impact of a 4-week mindfulness meditation program on perceived stress and self-compassion among nursing healthcare professionals during the COVID-19 pandemic.	26 nurses (17 in the intervention group and 9 in the control group)	pre-test/post-test design	A 4-week mindfulness meditation program using the Calm App, with participants asked to engage in a daily 10-min mindfulness practice.	4 weeks	The intervention group showed statistically significant improvements in two key constructs: confidence in handling problems (PSS) and maintaining a balanced perspective on life situations (SC). Other constructs demonstrated positive trends, though they were not statistically significant.
Corrigan et al. [39]/2024/United Kingdom	To assess the feasibility and potential benefits of a brief online compassion-focused intervention for ICU nurses during the COVID-19 pandemic.	26 nurses	pre-test/post-test design	A 4-week, online self-compassion intervention using Compassion Focused Therapy principles.	4 weeks	Improvements in compassion, burnout, trauma, and emotional climate were observed among completers, with higher engagement among those with pre-existing self-compassion.

**Table 5:** Synthesis of interventions.

Interventions	Outcomes	Assessment Tool	References
Mindfulness-Based Intervention Programs			
Mindfuless-based stress reduction (MBSR)	occupational burnout	The 22-item Maslach Burnout Inventory-Human Services Survey (MBI-HSS)	Mäkinen et al. [32]
	well-being	The WHO (Five) Well-being Index (WHO Five)	
	participants’ immediate experiences	Pulse Surveys	
Guided Respiration Mindfulness Therapy (GRMT)	perceived stress	The Perceived Stress Scale-10 iii (PSS-10)	Shu-Chen et al. [29]
	mindfulness	The 15-item Mindful Attention Awareness Scale (MAAS)	
	Self-Compassion	The Self-Compassion Scale (SCS)	
Mindful self-care and resiliency (MSCR)	compassion satisfaction/compassion fatigue	The 30-item Professional Quality of Life Scale–Version 5 (ProQOL-5)	Slatyer et al. [27]
	Depression, Anxiety and Stress	The Depression, Anxiety and Stress Scale (DASS21)	
	Self-Compassion	The Self-Compassion Scale (SCS)	
	Resilience	The Connor-Davidson Resilience Scale (CD-RISC10)	
	Self-Efficacy	The General Self-Efficacy Scale (GSES)	
	Well-being	The WHO (Five) Well-being Index (WHO Five)	
Mindfulness-based intervention (MBI)	Occupational Burnout	The 22-item Maslach Burnout Inventory-Human Services Survey (MBI-HSS)	Sahar et al. [31]
	Mindfulness	The Five Facet Mindfulness Questionnaire (FFMQ)	
	Self-Compassion	The Self-Compassion Scale (SCS)	
Compassionate-Based Interventions			
Mindful Self-Compassion (MSC)	Mindfulness	The Five Facet Mindfulness Questionnaire (FFMQ)	Martinet et al. [33]
	self-Compassion	The Self-Compassion Scale	
	Compassion Satisfaction/Compassion Fatigue	The 30-item Professional Quality of Life Scale–Version 5 (ProQOL-5)	
	Resilience	The Connor-Davidson Resilience Scale (CD-RISC10)	
Self-Compassion for Healthcare Communities (SCHC)	Self-Compassion	The Self-Compassion Scale (SCS)	Franco et al. [35]
	Compassion	Compassion Scale (CS)	
	Compassion Satisfaction/Compassion Fatigue	The 30-item Professional Quality of Life Scale–Version 5 (ProQOL-5)	
	Mindfulness	The 15-item Mindful Attention Awareness Scale (MAAS)	
	Depression, Anxiety and Stress	The Depression, Anxiety and Stress Scale (DASS21)	
	Resilience	The Connor-Davidson Resilience Scale (CD-RISC10)	
Compassion Focused Therapy (CFT)	Compassion Satisfaction/Compassion Fatigue	The 30-item Professional Quality of Life Scale–Version 5 (ProQOL-5)	Corrigan et al. [39]
	Self-Compassion	The Self-Compassion Scale	
	Emotional Climate of Organizations	Emotional Climate of Organizations Scale (ECOS)	
Loving-kindness Meditation (LKM)	Self-Compassion	The Self-Compassion Scale (SCS)	Valluri et al. [26]
	Chronic pain	The Numeric Pain Rating Scale (NPRS)	
	Caritas Factors	The Caring Factor Survey–Care Provider Version (CFS-CPV)	
	Perceived Stress	The Perceived Stress Scale-10 iii (PSS-10)	
Emotional Management-Based Interventions			
the Bonny Method of Guided Imagery and Music (GIM)	Self-Compassion	The Self-Compassion Scale (SCS)	Shum et al. [28]
Integrated Emotional-Self Enhancement (IESE)	Self-Compassion	The Self-Compassion Scale (SCS)	Saikia et al. [30]
	Emotional Intelligence	GENOS Emotional Intelligence Inventory	
	Emotional Labor	Emotional Labor Scale	
Resilience, Insight, Self-compassion, Empowerment (RISE)	Insight	Brief Resilience Scale (BRS)	Sawyer et al. [40]
	Self-Compassion	Self-Compassion Scale–Short Form (SCS-SF)	
	Stress Mindset	Stress Mindset Measure–General (SMM-G)	
	Perceived Stress	Perceived Stress Scale (PSS)	
	Burnout	Maslach Burnout Inventory (MBI)	
	Resilience	Brief Resilience Scale (BRS)	

## Data Availability

Data are contained within the article.
